# “Too little, too late”: a qualitative exploration of the unique pain management unmet needs of young adults with migraine

**DOI:** 10.3389/fpain.2026.1783714

**Published:** 2026-06-04

**Authors:** Caes Line, Drüeke Melissa, Jordan Abbie, Burr Charlotte, Filzmoser Nicola

**Affiliations:** 1 Division of Psychology, Faculty of Natural Sciences, University of Stirling, Stirling, United Kingdom; 2 Department of Psychology, University of Bath, Bath, United Kingdom; 3 National Migraine Centre, London, United Kingdom; 4 Happyr Health, United Kingdom (affiliation at the Time of Study, but Happyr Health Ltd is Currently Closed )

**Keywords:** migraine, pain management, peer support, vocational goals, young adults

## Abstract

**Introduction:**

Whilst a sizeable portion of young adults experience migraine, little is known about the unique support needs of these individuals at the critical developmental timepoint of young adulthood. In this study we aimed to identify the unmet needs for pain management in young adults (18–30 years) who experience migraine and how pain management programmes can be adjusted to better meet those needs.

**Methods:**

Individual interviews and focus groups were conducted online with seven young adults who experience migraine pain (*N* = 6 female, *M*_age_ = 23.6 years) and eight clinicians providing pain management support to young adults with chronic pain, including migraine (representing psychology, anaesthesiology, nursing, physiotherapy & hypnotherapy). Qualitative data generated from both young adults and clinicians was jointly analysed using reflexive inductive thematic analysis.

**Results:**

Two main themes were generated: (1) Too little too late; and (2) The challenges of finding their tribe. The first theme highlights that young adults would benefit from holistic, interdisciplinary treatment options tailored to their individual needs early on in their chronic pain journey, but access to these services is limited and offered too late in the process. The second theme represents the increased need for support in relation to social life and making vocational decisions, which could be met through peer support groups and positive representation.

**Discussion:**

These insights underscore the need to improve access to holistic, interdisciplinary and personalised care for young adults living with migraine which incorporate online and peer-based support approaches to address issues related to maintaining social connection and career planning.

## 1 Introduction

Chronic pain, defined as persistent or recurrent pain lasting longer than three months (1), is an alarming and pervasive health condition. Recent studies suggest that approximately one in nine young adults (aged 18–30) report experiencing chronic pain (2), with the most common pain type being headaches, including migraine. Migraine is a type of primary headache disorder (4), with symptoms that are often very intense and debilitating. Importantly, in the latest classification of chronic pain by IASP (2019), chronic migraine, has been recognised as a primary chronic pain condition [i.e., a pain condition in and off itself, without an underlying diagnosis, that is multifactorial in nature (5)]. Migraine and headache disorders are a global public health concern and the second most common cause of disability worldwide (6). An estimated 52% of individuals worldwide have a headache disorder, with 14% of those experiencing migraine [of which 8% experience chronic migraines (7)] and over 1 billion individuals experiencing migraines per year (6, 8). Prevalence rates in the USA, revealed that 18.7% of 18–44 years olds experience migraine (9), with recent worldwide lifetime incidence and prevalence data further confirming that young adults are particularly affected by migraine: the age group 10–14 years had the highest incidence rate, with a peak in prevalence between 30 and 34 years (8). Since migraine typically persists into adulthood, it is important that young people have the appropriate support and tools to help them manage their migraine and ameliorate its impact on their health, quality of life and daily functioning (10).

Importantly, the transition from adolescence to early adulthood is a critical developmental stage, characterised by essential transitions, such as progression to college or employment, living independently from parents and exploring more significant romantic relationships (11). The importance of this transition, especially in the context of chronic conditions, was identified by Sawyer and colleagues (12), who advocated for an increase in the upper age limit of paediatric services (up to age 24) due to upwards trend of disease burden across adolescence. The experience of chronic pain, including migraine, can significantly impede young adults' progression towards this normative level of independence (13, 14). Beyond the far-reaching consequences of chronic pain on autonomy, physical and psychosocial well-being (14), chronic pain can be detrimental for their future career prospects. Recent studies provide evidence for lower rates of college attendance/graduation and employment (including being more likely to be in a job that does not match their desired long-term career goals) in young adults with chronic pain (13, 15). For individuals with migraine specifically, Burch and colleagues (9), report an unemployment rate of 40% in adults experiencing migraine. For young people with chronic pain, including migraine, early adulthood also features the transition from paediatric to adult healthcare services, which is a complex process involving a large variety of stakeholders (i.e., parents, young adults and interdisciplinary healthcare providers), with many young adults experiencing significant barriers in this transition, leading to lapses in their care (2). Further complicating the situation, there is no consensus concerning what a successful transition looks like.

To better understand these healthcare transition challenges, Murray and colleagues (3) developed a Healthcare Transition Framework for young adults with chronic pain, which identifies the impact of and interaction between various socio-contextual factors (e.g., socioeconomic status, pain-related interference, neurocognitive status and family functioning) and healthcare transition processes (e.g., transition readiness, self-management skills, relationship with healthcare providers) on successful transition outcomes (e.g., regular use of self-management skills and engagement with adult care).

Despite this significant prevalence and impact of migraine on young adults' daily life, a thorough exploration of their unique support needs to manage their pain experiences has been largely overlooked, under-researched, and under-treated (2). Many conventional, evidence-based pain management approaches are either targeted at or tailored to paediatric or older adult populations (10, 16, 17). Hence the unique developmental, psychosocial, and physiological needs of young adults, e.g., a lack of social recognition of migraine pain in their peer group and/or workplace environment, are being neglected in current pain management programmes (14, 17). This not only leaves young adults isolated, needing to take on an advocacy role to get their needs met, but also a gap in our understanding of the clinical meaningful outcomes of pain management programmes for young adults (14, 17). To address this knowledge gap and develop interventions which meet the unique developmental needs of young adults, there is an urgent need to work closely with young adults with lived experiences of accessing support for their migraine pain.

Consequently, the main objective of this study was to identify the unmet needs for pain management in young adults (aged 18–30) experiencing migraine and how pain management programmes can be adjusted to meet those needs. To accomplish this and gain a comprehensive, multi-informant view on the challenges faced by young adults, individual and group interviews were held with young adults who experience migraine and interdisciplinary clinicians with at least one year of experience in supporting young adults with chronic pain management, including migraine (further referred to as clinicians).

## 2 Materials and methods

### 2.1 Participants

Potential young adults were recruited, using convenience sampling, via social media (Twitter/X) posts, the newsletter of The National Migraine Centre, and via charities (e.g., Pain Concern forum posts). A total of seven young adults with chronic migraine, aged 18–30 years old participated in the study. Young adults were mostly female (*N* = 6 females, *N* = 1 male) and on average 23.57 years old (range: 21–30; SD = 3.87). A total of *N* = 5 were currently in education, with *N* = 2 currently employed full time (both had received a higher education degree). The average duration of pain experiences was 6.8 years (range: 2 years to 10 years). A total of *N* = 2 participants had never received official treatment for their pain, *N* = 1 had received treatment in the past but were not receiving treatment at the time of taking part in the research, and *N* = 5 were currently receiving treatment. For those receiving treatment, a wide variety of specialists were delivering treatment, comprising general practitioners/GPs (*N* = 4), neurologists (*N* = 5), rheumatologists (*N* = 1), psychologists (*N* = 1) and occupational therapists (*N* = 1).

Clinicians were recruited, using convenience sampling, via social media (Twitter/X) posts, the newsletter of The National Migraine Centre, and via professional Listservs (e.g., Paediatric pain and Paediatric Pain Travel Club). A total of eight clinicians participated, most of whom were female (*N* = 7 females, *N* = 1 male) and on average 42.25 years old (range: 29–47; SD = 7.50). All were working full time and the average years of experience of working with young adults with chronic pain was 12.69 years (range: 3–26; SD = 9.76). A wide spread of disciplines was represented amongst the participants (*N* = 3 psychology, *N* = 2 anaesthesiology, *N* = 1 nursing, *N* = 1 physiotherapy, and *N* = 1 hypnotherapy).

Guided by sample sizes in similar previous studies (17), and the principles of information power (18), we believe this sample size is adequate to answer our research question as our study aim had a narrow focus with a rather homogenous target sample of participants with similar characteristics (e.g., UK—based young adults with migraine). Furthermore, during the data collection it became clear that the communication exchange between the researchers and participants was clear and strong. Consequently, we believe that we obtained strong and dynamic data, reflecting the highly dialogue quality between the interviewers and the participants. Combined, the high quality and rich narratives, narrow focus and homogenous target sample are congruent with the need for a smaller sample size in relation to the principles of information power (18).

### 2.2 Procedure

Ethical approval was received by the General University Ethics Panel (GUEP) at the University of Stirling (GUEP number 13563). Interested young adults or clinicians could click a link in the social media post or email that would allow them to read through the detailed information sheet online (using the secure Online Survey JISC platform (https://www.onlinesurveys.ac.uk). After reading the information sheet, participants provided consent to take part and were requested to complete some demographic details (e.g., age, gender, education level, occupational status, pain, and pain treatment experiences (for young adults) or years of experience in current occupation (for clinicians) and leave their email address so the research team could contact them to schedule an interview. Interviews/focus groups were scheduled based on the preference of the participants for an interview or focus group, as well as the researchers and participant's availability. All interviews/focus groups took place online, using the secure Microsoft Teams platform. Before the start of each interview/focus group, participants provided verbal consent to participate and for the conversations to be recorded. After each interview/focus group, participants were emailed a debrief form providing more details about the study and links to various support services, as well as a £20 gift voucher to thank them for their time.

### 2.3 Interviews and focus groups

While individual interviews are conducted one on one to obtain the personal narrative from participants, focus groups offer the unique opportunity to observe interpersonal dynamics between the participants (e.g., observing expressions of validation and connection through similar care experiences being shared or stimulating franc, open discussions that explore different opinions) (19). To ensure that participants remained at the heart of the study and that the study was as inclusive as possible, participants were given a choice between individual interviews and focus groups based on their preference. Especially given the sensitivity of the topic being discussed in this study, providing the participants with the choice of a focus group or individual interview would ensure that all voices and experiences—including minority voices—are shared and explored in equal amount of depth (20).

A semi-structured approach for both the interviews and the focus groups was deemed appropriate to our study's objective, as the scheduled questions guides the direction and content of the discussions but also enables the interviewer to follow-up on topics of interest that occur within the discussions through request of elaboration or follow-up questions (21). The scheduled questions explored current care pathway, what's missing in current care, and the current challenges with pain management. Prompts were used to encourage participants to expand on answers, provide more detail where required and to enable the researcher to follow interesting leads. Please see Table 1 for a full list of the scheduled questions. The scheduled questions were co-designed between the research team and the Happyr Health team, which includes a young adult living with migraine. All the focus groups and interviews were also conducted collaboratively, with one researcher and one person from Happyr Health team present at any focus group or interview.

**Table 1:** Overview of interview questions.

Young adults	Clinicians
What pain care do you receive at the moment and how? What are you missing in pain care? What are your biggest challenges with your pain? Tell us about a success story with your pain. What happened? How did you feel about it? Tell us about a success story with a patient. What happened? How did you feel about it? What do you enjoy most about working with young adults with chronic pain?	How would you care for a patient if you could take all the time and financial resources you want? What are you missing in current pain care? What's your biggest challenge in working with young adults with chronic pain? Is there a gap between what you think is part of good pain care vs. what you can deliver?

Data for young adults was collected across two focus groups comprising three young adults, and one individual interview. Clinicians took part across two focus groups with group comprising two clinicians each. An additional four clinicians completed individual interviews. All interviews and focus groups took place online, using Microsoft Teams. To confirm the identity of the participants and manage fraudulent participation, individuals were requested to turn their video on at the start of the interview and answer some demographic questions (to match them to their responses on the online form). After this identity check, participants were allowed to turn their camera off if they wished to do so. The interviews with young adults lasted on average 53 min (range from 38 to 66 min) and took place between April 2023 and May 2023. Semi-structured interviews or focus groups with clinicians lasted on average 50 min (range from 36 to 57 min) and took place between April 2023 and June 2023.

### 2.4 Ethical challenges

In recent years it has been acknowledged that imposter participants can be an expected risk for online qualitative research [see for example (22). Consequently, the key ethical challenge experienced during this study was filtering out imposter participants. Following various guidance publications [e.g. (23)], the following steps were taken before inviting interested participants onto focus groups/interviews:

- Basic demographic data and chronic pain data was gathered after signing the consent form, which was used as an eligibility check before interview/focus group invitations were send as well as an identification check at the start of the interviews/focus groups (see above).
- Telephone numbers were gathered to verify their authenticity.

Furthermore, the research team was wary of expressions of interest that came from peculiar email addresses, were brief and only focused on confirming eligibility and renumeration, as well as large number of expressions of interest arriving within a short timeframe (e.g., a data surge). No further contact was made with expressions of interest that were identified to be from imposter participants.

Despite these careful considerations, during the identification process of one of the focus groups with three young adults, two young adults were deemed to comprise imposter participants as their introduction did not match the basic details collected in the online demographic form. In addition, requests for further clarification were answered in a general manner, reflecting their lack of lived experiences with chronic pain. Consequently, this focus group was disbanded and the remaining genuine participant agreed to continue within an individual interview format.

### 2.5 Data analyses

All the interviews and focus groups were recorded and transcribed, to facilitate inductive reflexive thematic analysis (Reflexive TA) (24), using NVivo software, a qualitative data analysis software (25). Reflexive TA is an established qualitative approach suitable for our study objective as it is theoretically flexible and emphasises intentionality and critical appraisal of the data. Indeed, in Reflexive TA, the researchers' unique perspectives and lens are seen as central and valuable in interpreting the data and generating the themes [see reflexivity statement below (24)]. An inductive, data-driven approach, driven by a critical realist perspective, was adopted to analyse the data to facilitate a detailed exploration of young adults' and clinician's experiences with pain management (26). Critical realism is a perspective characterised by assuming an objective reality exists, but this reality is mostly invisible and only accessible through the subjective inference of experiences and research data (24).

LC led the Reflexive TA for the young adult interviews, while MD led the Reflexive TA of the clinician interviews. Both worked iteratively through the six phases described by Braun and Clarke (24]: data familiarisation, inductive coding, initial theme development, theme review, theme definition, and write up. Both LC and MD undertook repeated reading of the data to support immersion and reflexive engagement, documenting initial observations and analytic questions. Coding was conducted inductively, attending closely to participants' accounts rather than applying predefined categories. Codes were continually refined as analysis progressed, allowing interpretations to develop across the dataset. LC and MD discussed the developed codes when they both completed the inductive coding stage and before moving on to the initial theme development state. These discussions revealed a strong overlap between the identified codes in the narrative of young adults and clinicians. Consequently, it was decided to merge the developed codes from this stage onwards, and present a unified insight. In the subsequent initial theme development stage, themes were generated through identifying patterns of shared meaning across codes and considering their relevance to the study objectives. Theme development was iterative rather than linear, with LC and MD moving between raw data, coded extracts, and developing themes to examine coherence and distinctiveness. Throughout the later analytical stages, developing themes were discussed by LD and MC on several occasions with the wider research team. These discussions supported reflexive interrogation of interpretations, consideration of alternative explanations, and ensured that themes captured participants' experiences while remaining coherent and credible. This iterative process of development and refinement resulted in the generation of two final themes which are clearly written up in the results section alongside accompanying anonymised data extracts.

### 2.6 Quality in qualitative research

It is important to consider the quality of the research, from project inception to write up of the results in relation to quality criterion. The Reflexive Thematic Analysis Reporting Guidelines (RTARG) have been followed in writing up of the study rationale, procedures, analyses and interpretation. These guidelines particularly ensured that we have a strong rationale for 1) our research question, 2) participant selection and sample size, 3) the use of both interviews and focus groups, and 4) triangulation of the young adult and clinician data. Furthermore, the guidelines supported the provision of a detailed description of the reflexive thematic analyses process we implemented, resulting in a balanced and coherent narrative between our analytic perspective and participant quotes.

Addressing quality in more detail, we first considered confirmability (i.e., the extent to which the results are grounded in the data) (27), by including quotations throughout the description of the themes, and importantly, across the full range of participants (ensuring all voices were heard and represented). Second, we ensured that our interpretation of the data was grounded in examples (28) by providing data (quotations) throughout our written account to support our interpretation. Third, we ensured that the researchers were active in the process of the analytical processes, reflecting how themes were generated through active researcher engagement with the data and the data itself. This is further supported by our engagement with reflexivity throughout the research project.

Reflexivity on the active role of the researchers throughout the research and analyses was achieved in a variety of ways (24). Importantly authors (LC, MD and NF) were actively involved in conducting the interviews and focus groups. As LC and MD also conducted the analyses, they reflected upon this active involvement with data collection and were mindful of any potential influences these interactions with the participants could introduce throughout the analyses process.

Lastly, it is of importance to reflect on the position of the researchers in relation to the participants. LC is a white, late 30s woman and parent, with no history of chronic illnesses, who has an established research career with over a decade of experience in conducting scientific studies of paediatric chronic pain. Her research focuses mainly on understanding the social and psychological influences shape young peoples' pain experiences across their development (from infants to young adults). MD is a white woman in her mid-20s with a diagnosed chronic illness, studying psychology at undergraduate/postgraduate level with no previous experience conducting scientific studies of young adults with chronic pain. Consequently, the first author (LC) is not part of either group from which the data originates, while the second author (MD) has similarities in terms of age and chronic illness experiences with the young adults. However, LC has had previous professional interactions with some of the clinicians who participated in the study. With respect to the entire author team, NF is a white, young adult female who has been living with migraine since adolescence and was at the time of the study the co-owner of Happyr Health (however currently Happyr Health is closed). CB is a white, 40-year-old female and parent with a family history of migraine. Charlotte is the operations director for the National Migraine Centre and has worked there for over 10 years and in this role supports young adults with migraine. AJ is a white woman with an established career in the field of paediatric pain and in particular, qualitative methods. AJ has no personal experience of pain but notes that she is a parent of adolescents (not young adults). AJ's work is centres on the ideas around validating individuals' pain experiences and exploring better ways to support children, adolescents and their family members in the context of living with chronic pain. In sum, it is important to acknowledge that the entire research team consisted of white females across young and middle adulthood, with a shared motivation to improve pain care for young people, including young adults. While this collective perspective matches the demographic of—and hence enhanced the connection with—the participants, this could represent a source of bias in the interpretation of the findings.

## 3 Results

An inductive reflexive thematic analysis generated 2 themes that reflected the current unmet needs in pain management support for young adults with migraine, with associated suggestions of how to overcome those gaps. In line with Reflexive TA guidelines, these themes were generated to represent an independent, central, organizing meaning (29). Themes comprised: 1) Too little too late and 2) The challenges of finding their tribe. Themes are described individually below with quotations provided to support our interpretation. Pseudonyms have been used throughout to ensure participant confidentiality.

### 3.1 Too little, too late

Timely and appropriate treatment was a notable concern for young adults with migraine and clinicians, with all participants describing how they felt that holistic interdisciplinary treatment was the most important treatment for migraine. By holistic, interdisciplinary treatment we mean non-medical psychosocial interventions, such as lifestyle recommendations, relaxation/meditation and cognitive behavioural techniques, alongside medical treatment). However, the availability of such interdisciplinary approach is inconsistent due to a lack of resources, funding, and specialized staff in interdisciplinary teams, leading to long waiting lists and limiting the range and duration of interventions available: *“Yeah, we certainly don't have an adequate physical setup that I think is particularly appropriate for young people and we certainly don't have enough staff, we have you know a good variety of people that we can tap into, but not at the frequency we need, and no one has enough time.” (Mathilda, clinician, anaesthesiologist)*. Consequently, young adults experience gaining access to more specialist, interdisciplinary care as a long, exhausting processes, described by Mia (young adult, 25) as *“a steep uphill climb doing the research and fighting GPs for appointments and waiting times*”. Indeed, clinicians recognised that interdisciplinary treatment often *“comes too late in their journey.” (Stella, clinician, physiotherapy*), and expressed how earlier specialist interventions are crucial to meet the young adults' needs, thereby avoiding unnecessary medical treatments and the development of a chronic pain identity. For instance, Sarah (clinician, psychology) expressed how pain “*can become the identity before [receiving interdisciplinary treatment]*”, with one of the most enjoyable parts of her role being “*able to see somebody go from living a life that's totally focused on their pain to living a life that has more fulfilment and more meaning than just being focused on pain*.”

Importantly, delayed access can cause a reluctance or reduced motivation in patients towards non-medical approaches, like psychological treatments, and thereby negatively impact the success of holistic, interdisciplinary treatment approaches. By the time a referral to psychological treatment is offered, patients are often so fed up with the lack of success with medical treatments that they are willing to just try anything. Such an attitude is not the best starting point: *“There are lots of research studies out there saying how strong expectation actually influences the outcome of a treatment. But then in the context of chronic pain, I always feel that must be quite difficult because, by the time they come to a multidisciplinary treatment centre or your clinic, they already have gone through so many negative experiences that their expectations are so low that they're just trying anything.” (Nicolas, clinician, hypnotherapy).* Despite the delayed access, the one young adult who had had access to psychological treatment, reflected on how this *“whole look at life”* approach was most helpful to overcome their fear of a flare-up of pain, as well as identifying how lifestyle changes could be implemented to help manage their pain: “*A lot of mindfulness meditation, really change and really looking at my lifestyle and what factors were at play, whether my job was too stressful, if I wasn't having enough downtime and hobbies, things like that*.” (*Michelle, young adult, 30*).

A key factor that is exacerbating this challenge of receiving timely holistic, interdisciplinary care is the lack of patient-centred care offered by general practitioners (GPs), characterised by appointment durations (e.g., maximum 15 min) too limited to understand complex needs, a lack of knowledge on migraine management, and a primarily medical-centric approach. This medical-centric approach was remarkably reflected in the narratives of the young adults' pain management journeys, which mainly focused on their—mostly unsuccessful—experiences with medications prescribed by GPs: *“I've tried a lot of different pain medications, starting with just low-standard paracetamol, ibuprofen, naproxen, amitriptyline, propranolol, and all sorts of variety of cocktails of drugs. Haven't really ever found anything that works for me.” (Michelle, young adult, 30). “Improving the basic knowledge of GP's” (Phoebe, clinician, Nursing)* is crucial given primary care is the first port of call for pain management support and serves as the gatekeepers for further referrals. Currently, the lack of adequate support from GPs forces young adults with migraine to become their own pain detective, i.e., doing their own careful research and making deductions about the best approach to manage their symptoms or which specialist care to pursue.

Despite immense intrinsic motivation, this pain detective role comes with its own challenges. Young adults reflected how the nature of migraine symptoms (e.g., finding it difficult to focus on a screen) made such self-directed information gathering taxing and could even further worsen their symptoms: “*part of the issue is having chronic daily headaches are trying to read stuff on a screen does also give me a headache, so it's quite difficult doing my own research.” (Alison, young adult, 25*). Furthermore, while young adults were aware that they should only rely on resources that they felt were trustworthy, like NHS websites, public lectures, and the National Migraine Center website, they often found themselves navigating a sea of irrelevant and untrustworthy information before finding the right support: *“It can be quite overwhelming when you just put a Google search in and there's kind of a lot of websites that you don't really know if they're trustworthy.” (Hannah, young adult, 20).* Consequently, young adults are tasked with the additional responsibility to make decisions about the credibility of the abundance of information available, before being able to judge the suitability and impact of the information for their situation, thereby potentially further delaying their ability to access evidence-based care.

To reduce the burden of weeding through and assessing the credibility of a large variety of informational sources to help them manage migraine-related pain, young adults expressed the need to develop an online platform that centralizes evidence-based information. This information should cover a wide range of interdisciplinary treatment options (e.g., stress management, adjusting lifestyle, mindfulness, yoga, sleep) and local services, presented in a youth-friendly, easily navigable interface: *“I think having like tab/length to sort some kind of information so we don't have to do all the research—well, still do research, but like in the sense of won't have to do as much and won't have to go through lots of sort of useless sorts or untrustworthy information” (Alison, young adult, 25**)***. Such a centralised platform would be particularly useful to ensure timely access to credible information on evidence-based alternative treatments. Indeed, young adults expressed how an important part of their pain detective role was electively trying alternative pain management approaches to find something that could relieve migraine pain (even if just temporarily). For example, cannabis, acupuncture and tinted glasses for light-sensitive migraine were explored with varying degrees of success. One of the most common and more long-term successful alternative treatments reflected upon was cold treatment, either in the form of cool'n'sooth or wild swimming: *“Another thing that I find really positive for my pain is I'm really into wild swimming like swimming in the river all around the year. So I don't know. I find the like cold helpful.” (Evelyn, young adult, 21)*.

While young adults identified a centralised platform of credible information as a key avenue to overcome the current challenges in primary care, clinicians rather advocated for more “*skilled practitioners in primary care” (Stella, clinician, physiotherapy)*, thereby revealing a disparity between what young adults and clinicians value and wish to explore as potential solutions. Indeed, unanimously clinicians called for a comprehensive reform in primary care's approach to pain management, envisioning this as a pathway to more effective, patient-centric treatments for chronic pain: *“It's the same with chronic pain as it's everywhere and it's everyone's business. And we even know in university programs, often chronic pain is something that is sort of tacked on at the end. You learn about pain and then you learn about chronic pain and you know, we know one in 5 GP consultations are around pain. So, the first person you might see about your pain doesn't necessarily have the skills. Obviously, they have the potential to have the skills, but it's not part of their remit to be able to support the person in the way that we know is effective. And so I guess bringing in those skills into primary care in the form of OT's or physios or nurses that, you know, can support people early on.” (Stella, clinician, physiotherapy).* This quote illustrates how education about pain for healthcare providers also comes a little too late, and is rather tacked on at the end instead of being a key element. Hence, pain education for healthcare professionals needs to be more central and improved to ensure timely and appropriate pain treatment for young adults.

### 3.2 The challenges of finding their tribe

Whilst migraine pain impacted numerous domains of young adults' lives, participants described that the most salient impacts for them occurred in their social and vocational (education and work related) aspects of their lives, highlighting the need for more support and better understanding in these key areas by both their social circles as well as healthcare professionals. Unfortunately, receiving or providing age-appropriate support in these two areas was deemed challenging, often resulting in a mismatch between young adult's needs and the ability of clinicians to meet those needs. This mismatch leads young adults on a challenging path to find their tribe, i.e., peers with similar chronic pain experiences, who truly understand them and could offer meaningful support and advice.

The mismatch related to vocational opportunities was particularly highlighted as challenging, leading to a pervasive sense of being lost in decision-making about their future and career in young adults. For instance, young adults reported how the unpredictability of migraine pain affects their educational aspirations and career prospects. Many are struggling to engage in education and/or work due to pain as *“workplaces in general and educational spaces, they're not made to be accessible, unfortunately.” (Hannah, young adult, 20)*. Consequently, young adults expressed a lot of worry around whether to adjust their career paths to accommodate their pain or how to request specific adjustments where necessary. Even those who had secure, suitable jobs or were able to pursue their desired careers face concerns about being perceived as unreliable employees and how to appropriately discuss reasonable adjustments for their migraine experiences with employers: *“I literally cannot work when I have them [migraine]. I just think it sort of makes me look bad and it won't help me with getting any promotions or any raises or doing any of the jobs I actually really want to do. I just sort of have to cope with a job that works around my limitations around my migraines.” (Alison, young adult, 25)*.

Clinicians recognised a mismatch in meeting young adult's need for support with worries around their futures by acknowledging their limited training and ability to guide these young adults in career decisions, admitting their focus on engagement with current education paths rather than exploring future goals: *“I don't talk to my young people about what job they're going to do. We're so often focused on education, and I do wonder whether there's a bit of a gap there. I do wonder how challenging it really is to then go into the workplace when you have been struggling to even get into school, you know, and how you navigate that and having somebody who you can see has managed that.*” *(Danielle, clinician, anaesthesiology).* In the absence of other suitable support tools, clinicians often resorted to sharing success stories of others with similar pain experiences as the only form of encouragement to ease young adults' worries about their future. However, as Sarah (clinician, Psychology) indeed identified, there is a limit to the impact of a clinician sharing stories of peers in similar situation, with the *“power of hearing it from somebody who either is currently going through it with the same or similar condition being very strong”.* These challenges reflect a crucial need for young adults with migraine to find their tribe, in order to foster feelings of connectedness and empowerment with like-minded peers who share their unique experiences.

The participating young adults had a large variety of experiences in finding their tribe and were often still on this journey. Some of the young adults had been fortunate enough to engage with group-based psychological pain management programmes, which they described as extremely valuable and a breakthrough in their understanding of their own pain and management journey. Such open conversations between peers with chronic pain (regardless of aetiology) allowed young adults to hear how others have incorporated self-management techniques in a very practical way within their own lives and gave inspiration to try those ideas out themselves: *“I didn't really get much at all from the 1 on 1 sessions. But yeah just being in a group with other people who understood definitely helped… It was a lot of things I already knew. But just having it applied to, you know, people around you who are experiencing the same things is just a lot more impactful than just, you know, having advice and then having advice that you know is a bit more. It just feels more practical when you have people around you that are experienced the same thing.” (Steven, young adult, 20).* These peer connections gave them a sense of belonging, thereby reducing feelings of isolation induced by the strained relations with their friends without chronic pain, who “*really do not relate to what you're going through” (Evelyn, young adult, 21).* This sense of belonging is of particular importance for young adults as they are often facing a large variety of transitions simultaneously (e.g., moving out and starting higher education or vocational training, transitioning from paediatric to adult healthcare), thereby risking further exacerbation of their feelings of loneliness and uniqueness in their pain experience: *“If somebody comes away to university and is living away from home for example or not a university but just moved away from home and then they begin to struggle more with their pain. They can become much more isolated than maybe in general an adult might be who already has established a family with friendship groups and so on.” (Stella, clinician, physiotherapy)*. Furthermore, another unique risk to increased feelings of isolation and loneliness in young adults is the lack of tailored inclusion of parental/caregiver support for young adults in adult services. While young adults are semi-independent, they still require substantial and flexible parental support, which is not appropriately addressed within adult services and could have a detrimental impact on their wellbeing: *“They're still really dependent on parents. But we don't have that kind of parental support here because they're young adults. And I think that is a downfall because it is really difficult for young adults who are living with daily chronic pain and, you know, chronic depression and anxiety to hold themselves accountable and maintaining some of the expectations of the program and the ongoing exercise and it's a lot that they have to do.” (Georgia, psychology)*.

While peer support could reduce these feelings of isolation, a key to success of peer support is the age-appropriateness. However, most current adult-focused peer support groups fail to meet this criteria due to wide diversity of different life stages of participants participating in these support groups, thus lacking the essential element of shared experiences: *“It's difficult and we don't have a specific pain management programme for young adults, so if they went, if they were in that 18–25 group, they would have to be into the adult pain management program and they are not going to relate to Doreen, who's 83 with her 20-year history of back pain.” (Mathilda, clinician, anaesthesiology)*. Such a lack of young adult focused peer support groups left many of the young adults feeling isolated and still in search of their tribe, especially if they experienced unconventional symptoms of migraine that did not seem to fit with the standard presentation: *“I know it's very common in women to have migraines, and I'm aware of that. I just don't feel like anyone experiences it like how you do and normalises it. It can be very isolating.” (Rose, young adult, 24)*.

Being able to offer peer support online was offered as a suggestion to allow for wider opportunities to forge relatable connections as well as be compatible with young adults' familiarity with digital connections and schedules (e.g., minimizing educational disruptions) and hence enhancing engagement: *“I think that there are a huge number of benefits to doing stuff digitally and online. Young people like it, they relate to it better.” (Mathilda, clinician, anaesthesiology)*. While young adult narratives raised no concerns related to online peer support, clinicians advised careful planning to ensure its effectiveness and safety, considering factors like age, condition, and mental state of participants as well as appropriate moderation of group sessions to keep the interactions positive: “*Cohorting the right people together, if it's group work that the aim is for, you know, similar ages and similar sort of conditions and making sure there isn't a reason why these people will probably benefit from not being in sort of group sessions.” (Mathilda, clinician, anaesthesiology).* Specifically, clinicians warned about how language use within online platforms is crucial to consider, as the interactions should avoid that young adults *“label themselves as always being the chronic pain patient” (Danielle, clinician, anaesthesiology)*. Therefore, group sessions were rather seen as an addition, not a replacement, of individual sessions: *“I think you do have to find some way to balance, you know, sort of the positive side of things as well as the potential negatives. Umm, so I think you do need even if it's somebody in the background, somebody that's just making sure that you haven't got the odds with this constant negative view.” (Phoebe, clinician, nursing).* Regardless of the approach towards and challenges with peer support, clinicians did highlight how being able to provide age-appropriate peer support could also to meet the—currently overlooked—needs of young adults who need more support than a primary care setting can offer, but do not require intensive interdisciplinary treatment: *“There are some young people who don't need the intense kind of psychological support, but they need some psychological support within their rehab. I just wonder whether we've got extremes on either end, but we don't have the intermediate pain management programs that you have more of in adults. I think you need all three and I think we have a gap in the middle.” (Danielle, clinician, anaesthesiology)*.

In sum, finding their tribe was identified as challenging but particularly beneficial for young adult's wellbeing due to their unique developmental challenges (e.g., growing need for independence).

## 4 Discussion

This research set out to investigate the unmet needs of young adults with migraine and how pain management programmes can be adjusted to better meet those needs. Two main themes were generated and described as (1) too little, too late, and (2) the challenges of finding their tribe. Our findings reiterate the conclusions drawn by (14) and (17), which identified the isolating impact of chronic pain in young adults, fuelled by uncertainty about the future and a lack of tailored treatment options. The lack of tailored treatment options leaves young adults with a steep learning curve to advocate for themselves and a strong need to find their tribe, which fosters a meaningful connection with other young adults with migraine.

Our findings also align well with the Healthcare Transition Framework for Youth with Chronic Pain, developed by (2). In line with this framework, our findings support how the transition is a complex, evolving process that not only involves the young adult, but also requires individualised and flexible support from parents and clinicians to meet their unique needs (e.g., support for vocational choices). Specifically, our finding underscore how socio-cultural aspects such as access to appropriate (i.e., interdisciplinary care) treatment and differences in individual functioning (e.g., migraine symptoms making it difficult to focus on a screen for crucial information gathering) influence their transition process, especially their readiness to transition and the (mis)trust in clinicians' knowledge about their condition, which in turn impacts their transition outcomes. The narratives also align with the framework's position that successful transition doesn't necessarily mean engaging actively with adult clinicians, but rather calls for a holistic individualised evaluation of the young adult's wellbeing and satisfaction with care. This is particularly illustrated by the examples of postponing transition of medical care until other transitions (e.g., transitioning to university) have been satisfactory completed (3). However, our data supports a stronger role and involvement of age-appropriate peer support to navigate young adults through this complex pathway of transition.

### 4.1 Discussion of the themes

The first theme, “too little, too late”, identifies a consensus between young adults and clinicians' narratives with respect to early access to interdisciplinary, personalised approaches for migraine-related pain management is key, but lacking in the current services. An overhaul of how migraine symptoms are understood and managed within primary care settings were seen—by the participants—as an urgent requirement to improve support. It is important to acknowledge the healthcare context of the participants within this study, for whom primary care played a central role as a first contact point, for any referrals to further specialised care and remaining a central contact point throughout any treatment process. Furthermore, all young adults had some experience with specialised care and all clinicians worked within an interdisciplinary context. Consequently, the experiences and conclusions drawn might not be generalisable to other healthcare contexts where primary care plays a less central role. Regardless of this context however, the narratives further support the recommendation for holistic, interdisciplinary treatment (i.e., non-medical psychosocial intervention alongside medical treatment) as best practice for supporting migraine management (10). Indeed, both the narratives of young adults and clinicians revealed how primary care services—as a first point of contact for migraine symptoms- had a strong focus on a pharmacological approach towards pain management, with—according to our participants—limited knowledge and training about interdisciplinary management of migraine. Young adults articulately described how they often had to do their own research on alternative medications or approaches and then tirelessly advocate for these alternatives. Aside from the challenges to gather information themselves, it is also potentially dangerous as many young adults turn towards social media to find information that is not coming from an official source and thereby could be misleading and even dangerous (30). The medicalised approach at the start of their journey could explain why young adults focused primarily on the role and limitations of medication for treating their migraine, while in contrast, clinicians focused more on the psychological approaches. Despite large variations in their experience and impact of psychological treatment, most young adults did recognise the relevance of psychological support (10, 16, 31).

The second theme, “the challenges of finding their tribe”, encompasses the hardships that young adults face with managing their condition and the negative effect it has on their quality of life with a particular unique focus on their challenges to maintain an active social life and interrupted plans for their future, e.g., attending education and career planning. These findings further support growing evidence on how migraine could particularly negatively impact career paths, financial stability, romantic relationships and family planning (32). For instance, Murray et al. (13), found that young adults with a chronic pain condition are less likely to be in full-time employment overall, however, if employed they are less likely to be in a role that is beneficial for their long-term career goals. Disruptions in education is an experience that has been identified in the literature for adolescents with chronic pain conditions (14, 33), but our findings underscore how the lack of age-appropriate support, i.e., the lack of young adult peer support groups for managing migraine, can play a critical role in feeling unable to pursue future goals.

Indeed, in line with the explanations put forward by Murray et al. (13), a potential key barrier to adult services is not meeting the unique needs and preferences of young adults, such as supporting appropriate connections to other peers with chronic pain. There is growing support for the effectiveness of peer support for adolescents with chronic pain, identifying connecting with another adolescent or young adult with chronic pain improved their ability to manage pain and reduced feelings of isolation due to establishing a feeling of belonging (34, 35). Peer support also allowed adolescents to seek information from trained peer mentors with lived experience which is more credible than relying on unverified information on the internet (34). However, despite this growing evidence on the benefits of peer mentoring for adolescents with chronic pain, there is limited implementation and evaluation on the role of peer mentoring for young adults with chronic pain (including migraine). Within our study, the consensus of an online peer mentoring program was positive, especially amongst the young adults. Some clinicians did offer critical voices as they fear matching the wrong people together can lead to a decrease in psychological resilience and increase isolation from general society due to being too focused on the chronic pain as an identity (36). However, this could be counteracted by ensuring that the peer mentors have sufficient training and support in identifying such negative tendencies (36). In sum, these findings suggest that there is a need to design and evaluate tailored peer mentoring programmes for young adults with migraine.

### 4.2 Limitations

While our findings are valuable, they need to be considered in light of the limitations of our study. Our sample was limited in terms of gender and geographical representation, with most participants (including young adults and clinicians) being female and based in United Kingdom and the United States. Furthermore, the healthcare contexts represented within the study all include a central role for primary care to gain access to more specialised care, with all young adults having gained some access to specialised care at some point in their pain management journey and all clinicians working within an interdisciplinary context. Consequently, despite the gender distribution being reflective of the higher prevalence of migraine in females (6), generalisation of our findings to other genders, other types of chronic pain, and other countries with different healthcare provisions might be limited since health care varies greatly, even between western countries. Future research with samples representing a wider gender distribution of young adults and clinicians, including non-binary participants, and more diverse healthcare settings is warranted. Despite these limitations, the sample and methods used are also a strength as participants were recruited from a community sample and therefore represented a wide variety of experiences with their migraine and access to healthcare. The pain literature typically focuses on clinical samples, notably those in specialist tertiary pain services, hence these voices are often omitted from the literature. Furthermore, despite three clinicians being psychologists, the participating clinicians represented a good variety of disciplines involved in the care of young adults with chronic pain. Additionally, our study facilitated representation of the views of young adults and clinicians, something which most previous studies have failed to achieve due to a focus on only one of these populations.

### 4.3 Conclusion: implications for practice and research

Consequently, our findings offer a valuable insight into the lives of young adults with migraine underscoring the need for more accessible, interdisciplinary, and personalised care. The results provide a potential roadmap for future investigations and clinical practices emphasising a more tailored approach harnessing the benefits of online and peer-based approaches to pain management for young adults experiencing migraine. Crucially, our findings underscore how such tailored approaches need to address the unique challenges related to maintaining a social life and a suitable career young adults with chronic pain face, to make an age-appropriate and relevant positive impact on the lives of young adults experiencing migraine.

## Data Availability

The raw data supporting the conclusions of this article will be made available by the authors, without undue reservation.
